# A New Type of Hydraulic Clutch with Magnetorheological Fluid: Theory and Experiment

**DOI:** 10.3390/mi15050572

**Published:** 2024-04-26

**Authors:** Karol Musiałek, Ireneusz Musiałek, Karol Osowski, Artur Olszak, Aneta Mikulska, Zbigniew Kęsy, Andrzej Kęsy, Seung-Bok Choi

**Affiliations:** 1 Mechatronics Division, Jan Kochanowski University of Kielce, 25-369 Kielce, Poland; karol.musialek@ujk.edu.pl (K.M.); imusialek@ujk.edu.pl (I.M.); zkesy@interia.pl (Z.K.); akesy@op.pl (A.K.); 2 Faculty of Mechanical Engineering, Casimir Pulaski Radom University, 26-600 Radom, Poland; k.osowski@uthrad.pl (K.O.); aneta.mikulska@uthrad.pl (A.M.); 3 Łukasiewicz Research Network—New Chemical Syntheses Institute, 24-110 Puławy, Poland; artur.olszak@ins.pulawy.pl; 4 Department of Mechanical Engineering, The State University of New York, Korea (SUNY Korea), Incheon 21985, Republic of Korea; 5 Department of Mechanical Engineering, Industrial University of Ho Chi Minh City (IUH), Ho Chi Minh City 70000, Vietnam

**Keywords:** magnetorheological fluid (MR), hydraulic clutch operating with MR fluid, rotating magnetic field, rotational speed, particle sizes

## Abstract

This paper presents a new type of hydraulic clutch operating by means of magnetorheological (MR) fluids and the results achieved from both theoretical analysis and experimental measurement. A hydraulic clutch system with MR working fluid and a rotating magnetic field located was designed. The clutch was based on the principle of using a rotating magnetic field created by an alternating current electromagnet to set the MR fluid in motion. To test the hydraulic clutch with a rotating magnetic field, MR fluids were produced by our laboratory, consisting of solid iron particles of various diameters mixed with a silicone oil. With MR working fluid and a rotating magnetic core was designed. The rheological properties of the MR fluids were assessed on the basis of tests carried out with a Brookfield DV2T rheometer equipped with a magnetic device for generating a magnetic field. The characteristics of the hydraulic clutch were tested on a specially built test stand. It was found that the torque transmitted by the clutch increased with the rotational speed of the magnetic field and with a lower rotational speed of the beaker in which the working fluid was placed. It was also found that the greatest torque occurred with the working fluid with the highest iron content. Based on the analysis of the structure and characteristics of the clutch in which the magnetic field is used, it has been shown that the design of the developed clutch is similar to that of an induction clutch, and its characteristics correspond to the characteristics of the eddy current clutch. Therefore, the proposed new clutch with MR fluid and rotating magnetic field can be applied to stationary power transmission systems in a manner similar to an eddy current clutch.

## 1. Introduction

Mechanical clutches are one of mostly commonly used machine components. Clutches perform a number of tasks in drive systems, e.g., connecting and disconnecting shafts, overload protection, enabling or improving start-up, and equalizing loads during the operation of multi-motor drives. Due to this, perfecting clutch design is desirable in the development of machines, as it has a significant impact on the durability and functionality of machines. The driving and driven parts of the clutch are connected to each other with a connector—sometimes permanently, sometimes allowing slippage. In slipping clutches particularly, a fluid or a magnetic field may act as a connector. Hydraulic clutches whose connector is a fluid are built in viscous or hydrodynamic variants [1,2,3]. In typical viscous clutches, torque is transmitted as a result of friction caused by shear stresses in the working fluid located in the working gap formed by the driving part and the driven part of the clutch [4,5,6,7]. In classic magnetic clutches, the magnetic field is used to induce an electric current and thus generate magnetic forces between the torque-transmitting parts of the clutch [8].

The working fluids used in hydraulic clutches are usually hydraulic oils. However, clutches which use fluids with rheological properties controlled by electric current are also in use [9,10,11,12]. These fluids come in two types: magnetorheological fluids (MR) and electrorheological fluids. Those using MR fluids are more common, due to the fact that the shear stresses obtained in MR fluids are 20 to 50 times higher than in electrorheological fluids [13]. MR fluids are two-phase mixtures, or colloids, which change their rheological properties under the influence of a magnetic field [14,15,16]. The solid phase of the MR fluid consists of particles made of ferromagnetic materials such as cobalt, iron, iron alloys, and oxides of these metals. MR fluids whose solid particles have a diameter of several dozen nanometers are also known as ferrofluids or ferro-colloids. Their liquid phase is usually silicon oil, due to the fact that temperature has little effect on the rheological properties of this oil. MR fluids also contain chemical compounds which prevent solid phase sedimentation and particle aggregation. Apart from clutches, MR fluids are used as working fluids in various machine components [17,18], mainly in vibration dampers [19,20,21,22], electrohydraulic servo control systems [23,24], cantilever sandwich beams [25], and seals [26]. The main advantages that determine the technical use of these fluids are the ability to control shear stresses in the fluid using electric current and the short reaction time of the liquid to a change in the magnetic field (which allows for high dynamics of the controlled component).The relevant literature provides various theories regarding the impact of the rotating magnetic field on MR fluid placed in a beaker, under which circumstances theoretical considerations apply only to ferro-colloids. The forces which influence the phenomena occurring in ferro-colloids placed in a rotating magnetic field include magnetic interaction, intermolecular attraction, surface tension, centrifugal force, viscosity, friction, and gravity. The magnetic field is determined by the three following vector quantities: magnetic induction, $\vec{B}$; magnetic field intensity, $\vec{H}$; and magnetization, $\vec{J}$. These three are related by the dependency $\vec{B}=\mu_{0}\vec{H}+\vec{J}$, where the coefficient $\mu_{0}$ signifies the magnetic permeability of free space.

According to the authors of [27,28], there are two possible ways that magnetization, $\vec{J}$, of ferro-colloid particles reacts to a rotating magnetic field: the Néel mechanism and the Brownian mechanism. For smaller particles which cannot freely rotate within the fluid due to a greater resistance to movement, the magnetization vector rotates inside the particle. This manner of magnetization is called the Néel mechanism. The Brownian mechanism is related to the Brownian motion of solid particles within a fluid. The Néel mechanism occurs if the colloid consists of solid iron particles with a diameter smaller than 8.5 nm, while the Brownian mechanism occurs when the colloid consists of solid iron particles with a diameter larger than 8.5 nm. For ferro-colloid solid particles made of cobalt the limiting particle diameter is 4 nm, and for solid particles made of magnetite the limiting particle diameter is 13 nm [28,29]. However, it should be noted that this does not represent a general rule. Same ferro-colloids exhibit both Brownian and Néel relaxation in magnetic fluid [30].

Under the influence of a rotating magnetic field, larger ferro-colloid particles rotate freely within the fluid, with an angular velocity *ω* of the rotation of the magnetic field [31]. The base fluid in the immediate vicinity of the solid particles rotates as well, so each ferro-colloid particle becomes the center of a microscopic vortex. All particles rotate at the same angular velocity, *ω*, which means that there is no macroscopic movement of the ferro-colloid and only the outermost fluid particles (at the walls of the beaker) move. The rotating magnetic field affects the macroscopic motion of the ferro-colloid only when the magnetic field is non-uniform. However, for a larger volume of ferro-colloid to move, additional conditions must be met regarding the diameter and concentration of solid particles and the viscosity of the base fluid. A similar interpretation of the influence of the rotating magnetic field on the movement of ferro-colloid is presented in [32]. In a stationary magnetic field, the magnetization vector $\vec{J}$ is collinear with the magnetic field intensity vector $\vec{H}$. During the rotation of the magnetic field, the collinearity of these vectors can also be maintained for small solid ferro-colloid particles. Then, the magnetization vector $\vec{J}$ rotates freely with the solid particles in the base fluid. On the other hand, in large particles, when the magnetic field intensity vector $\vec{H}$ rotates with angular velocity *ω*, the magnetization vector $\vec{J}$ remains behind the vector $\vec{H}$ and is shifted by a certain lag angle. As a result of the lag angle, the vector product, $\vec{J}\times \vec{H}$, has a value other than zero and creates a torque, *M*, influencing the motion of particles in the rotating magnetic field. The solid particles rotate slower than the magnetic field because their movement is inhibited by the viscous forces of the fluid.

The authors of [33] proposed the “magnetic pole model”. Their assumption is that each volume element in a homogenous ferro-colloid can be assigned a magnetization vector $\vec{J}$ that is dependent on the concentration of solid particles and the lag angle α, in relation to the rotating magnetic field $\vec{H}$, dependent on the resistance caused by viscous forces. In the presence of a rotating magnetic field, the torque (resulting from vector product $\vec{J}\times \vec{H}$) induces rotation in the volume element, causing it to rotate with the rotation speed of the magnetic field *ω*. However, for bulk rotation of the ferro-colloid to occur, there must be in homogeneities in the ferro-colloid in the value $\vec{J}$ or the angle *α*. Then, a pair of mutually interacting magnets are created. As a result, one of these magnets pulls the other, which causes the whole volume of the ferro-colloid to rotate in the beaker with the speed of *ω*. In experimental research [31,32,33,34] on the behavior of various ferro-colloids placed in a beaker affected by a magnetic field rotating with an angular velocity *ω*, it was found that ferro-colloid solid particles rotated within the base fluid with an angular velocity of *ω*. This caused the base fluid to rotate around these particles at a speed lower than the angular velocity *ω*. Depending on the test conditions and the existing in homogeneities, magnetic field, magnetization, particle concentration, friction coefficient, and viscosity of the base fluid, it is possible that the entire ferro-colloid volume will move with an angular velocity lower than *ω*. In such cases, the ferro-colloid may rotate in the *ω* direction, rotate against the *ω* direction, or macroscopic vortices may occur. The rotation speed of the ferro-colloid depends on the angular velocity, *ω*; the magnetic field intensity; the location of the magnetic field; the size of the solid particles; and the magnetization of the solid particles. A thin ferro-colloid ring may appear near the beaker’s wall, rotating at an angular velocity much lower than *ω*. After adding an immiscible fluid to the ferro-colloid, one may observe the formation of a convex or concave meniscus as a result of the rotation of the magnetic field.

The effect of a rotating magnetic field on ferro-colloids was confirmed in [35], which examines the behavior of a ferro-colloid drop placed in an alcohol solution with simultaneously applied rotating and DC axial magnetic fields. Observing the placement of ferrofluid in a fluid reveals various distinctive patterns. The publications [36,37] present research on the behavior of ferro-colloids under the influence of a rotating magnetic field, utilizing a Brookfield rheometer. The rheometer spindle was immersed in a stationary beaker filled with a ferro-colloid. The beaker was affected by a rotating magnetic field generated by the stator winding of a three-phase induction motor. It was found that the torque acting on the rheometer spindle depended on the angular velocity of rotation of the magnetic field, the magnetic field intensity, and the magnetization of the ferro-colloid particles.

The study [31] also considers the possibility of increasing the efficiency of a hydrodynamic torque converter filled with ferro-colloid as a working fluid, placed inside the winding stator of a three-phase induction motor. However, as expected, there was no increase in the angular velocity of the output shaft of the hydrodynamic torque converter as a result of the impact of the rotating magnetic field on the ferro-colloid.

The present article introduces a novel construction concept for a hydraulic clutch incorporating a MR fluid (HCMR). This concept involves the application of a rotating magnetic field to induce the rotation of the MR fluid, consequently transmitting torque through friction against the beaker walls. The developed HCMR is at the “technology demonstration” stage.

## 2. Tests of the Hydraulic Clutch with MR Fluid

### 2.1. Construction of a Hydraulic Clutch with MR Fluid

**Figure 1:**
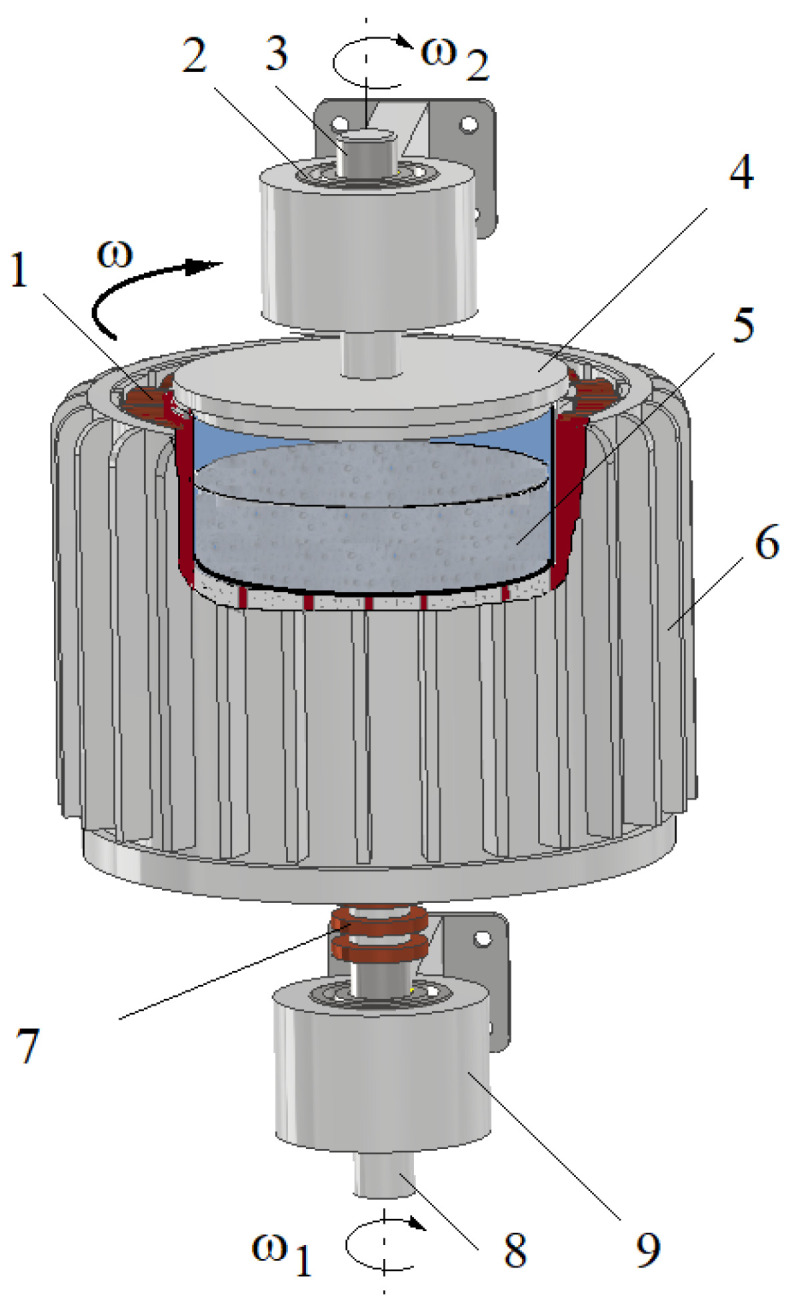
HMCR construction scheme: 1—coil, 2—bearings, 3—output shaft, 4—beaker, 5—MR fluid, 6—stator of a three-phase induction motor, 7—slip rings, 8—input shaft, 9—bearing housing.

On the input shaft of the HCMR there was an electromagnet that generates a rotating magnetic field. The electromagnet was built using the winding stators of a three-phase induction motor. Slip rings and brushes powered the electromagnet. Inside the electromagnet there was a beaker containing the MR fluid, connected to the output shaft. The input and output shafts were mounted in the housing. The HCMR structure is shown in Figure 1. The HCMR works in the following manner. A rotating magnetic field is created in the electromagnet. During clutch operation, the angular velocity *ω* of the magnetic field rotation *ω* is added to the angular velocity *ω*_1_ of the input shaft rotation. The magnetic field rotating with an angular velocity “*ω* + *ω*_1_” sets the MR fluid in rotation. As a consequence of friction against the beaker walls, the fluid rotates the beaker (and the output shaft connected to it) with an angular velocity *ω*_2_.

### 2.2. Tested MR Fluids

In-house manufactured fluids were used in the HCMR tests. The fluids consisted of solid iron particles in the form of spherical powder of various diameters, mixed with silicone oil. Laboratory sieves with mesh dimensions specified in the ISO 3310-1 standard [38] were used in order to isolate solid particles of the required diameter. Given the brief duration of the tests, no anti-corrosion additives or additives aimed at reducing sedimentation and aggregation of MR fluids were utilized. The data concerning the composition of the tested MR fluids are presented in Table 1. The fluids with the composition shown in Table 2 were prepared with a different weight concentration ratio, *φ*, of solid particles, marked with digital symbols. The full name of the MR fluid used in testing consists of the type of the fluid (expressing the size of the solid particles, A or B) and the symbol of the fluid (expressing the weight concentration ratio of solid particles, from 1 to 5). Thus, “A1” signifies a fluid whose particle size is 3.5 μm to 6.5 μm, and *φ* = 50%, as can be seen from Table 1 and Table 2.

### 2.3. Testing the Rheological Properties of MR Fluids

**Figure 2:**
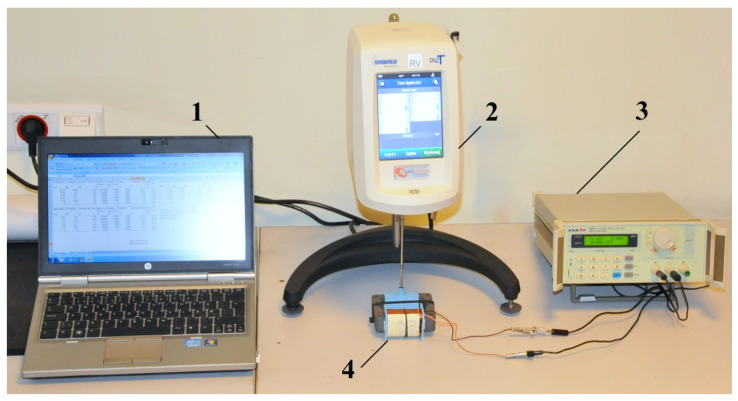
View of the station used for testing the rheological properties of the MR fluid: 1—PC, 2—rheometer, 3—electric power supply, 4—magnetic device.

In order to assess the rheological properties of MR fluids in the absence or in the presence of a magnetic field, a special test stand was constructed, as shown in Figure 2.

**Figure 3:**
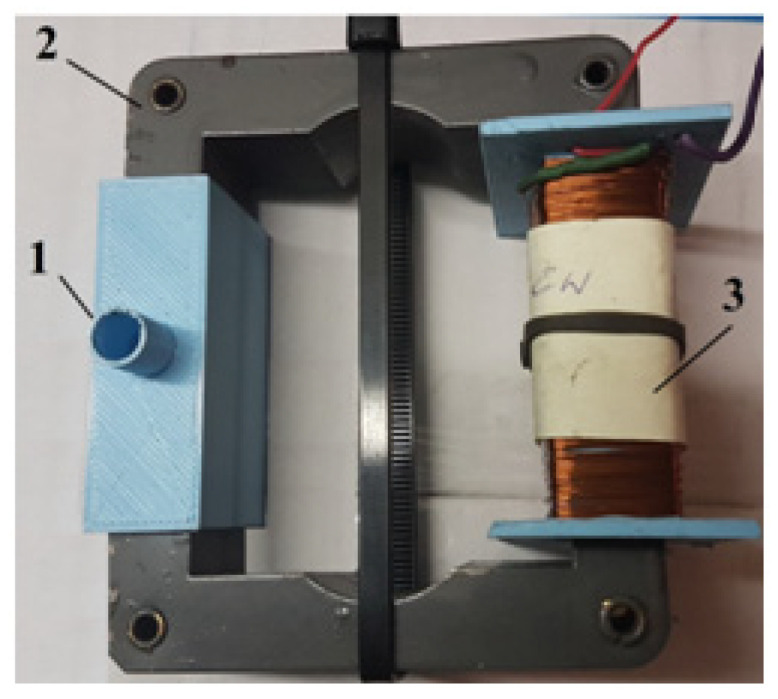
Magnetic device used to test the MR fluid: 1—beaker with MR fluid, 2—electromagnet coil, 3—magnetic circuit.

The test stand consisted of a Brookfield DV2T rheometer (Brookfield Engineering Laboratories, Inc., Middleboro, MA, USA) and an internally designed magnetic device with an electromagnet generating a magnetic field. The magnetic device is presented in Figure 3. The change in magnetic induction *B* in the MR fluid was achieved by supplying power to the electromagnet coil using an Array 3645A electric power supply with adjustable voltage. Measurements of magnetic induction in the MR fluid were performed using a Smart Magnetic Sensor SMS 102. The PC was equipped with Brookfield DV2T rheometer software (Firmware v 1.0.2), used to record the time course of the measured values. The waveforms were saved as files in the computer’s memory. In order to obtain the values of the torque, *M_r_*, loading the rheometer spindle, which were consistent with the rheometer manufacturer’s recommendations, several variants of the magnetic device design were created and tested. The test conditions and the dimensions of the magnetic device determined during the tests are presented in Table 3.

For MR fluids, the influence of temperature on its rheological properties is limited to the influence on the viscosity of the base liquid [39,40]. The magnetic properties of solid particles of MR fluid change at temperatures well beyond the temperature range at which MR fluid can be used [32].

**Figure 4:**
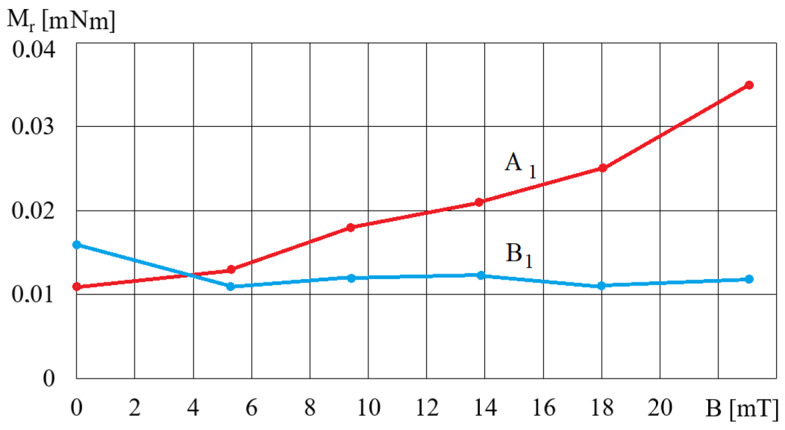
The dependence of the torque, *M_r_*, on magnetic induction *B* for fluids A1 and B1 and for *ω_r_* = 2 rad/s.

Figure 4 shows an example of the dependence of the spindle’s torque movement resistance, *M_r_*, on the magnetic induction, *B*, for fluids A1 and B1.

**Figure 5:**
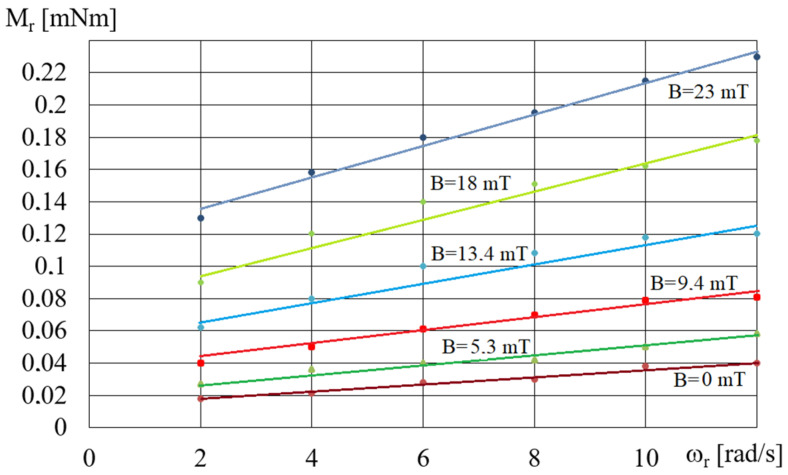
Dependences of the torque *M_r_* on *ω_r_* for fluid A5 and different values of *B*.

The comparison of the waveforms shown in Figure 4 with the waveforms obtained for MR fluids, such as MR 132 AD [40], shows that the waveforms obtained for the A1 fluid are the most similar, while the waveforms obtained for the B2 fluid are the most distant from the waveforms typical for MR fluids. The reason for these discrepancies is the homogeneity of the fluids during testing, which is related to the fluids’ structure. MR fluids containing small-diameter iron particles, such as fluid A1, are more homogeneous and under the influence of a magnetic field the iron particles do not move quickly, as the resistance to their movement in the oil is greater than the magnetic forces. Conversely, iron particles with a larger diameter, such as the ones in fluid B2, are more strongly attracted to the electromagnet poles and move much faster towards the beaker walls. This means that during tests the fluid containing more oil remains at the rheometer spindle, and therefore the influence of the magnetic field on the rotational movement of the spindle decreases. Figure 5 shows an example of the dependences of the torque *M_r_* on *ω_r_* for fluid A5 and various values of magnetic induction *B*.

As can be seen from Figure 5, in the case of fluid A, the value of the torque *M_r_* increases both with the increase in angular velocity *ω_r_* and magnetic induction *B*. As the concentration of iron particles in fluid A increases, a corresponding increase in the torque value *M_r_* is observed. As the magnetic induction increases, the angle of inclination between the approximating line and the positive *ω_r_*-axis increases. As the angular velocity *ω_r_* increases, so does the value of the torque *M_r_* in the absence of a magnetic field.

The relative measurement error of the measured quantities such as the angular velocity (*ω_r_*) and torque (*M_r_*) of the rheometer, voltage (*U*)and current intensity (*I*) of the electric power supply, magnetic induction (*B*), and fluid temperature (*T*) were less than ±1%. Due to the specific structure of the magnetic device, whose steel spindle rotates in a magnetic field, the occurrence of eddy currents was taken into account, as they have a braking effect on the spindle. Based on the results of tests conducted without MR fluids in the magnetic device’s container, the relative measurement error of the torque *M_r_* caused by eddy currents was estimated to be less than 5% over the entire range of tests.

### 2.4. Test Stand for the Hydraulic Clutch with MR Fluid

**Figure 6:**
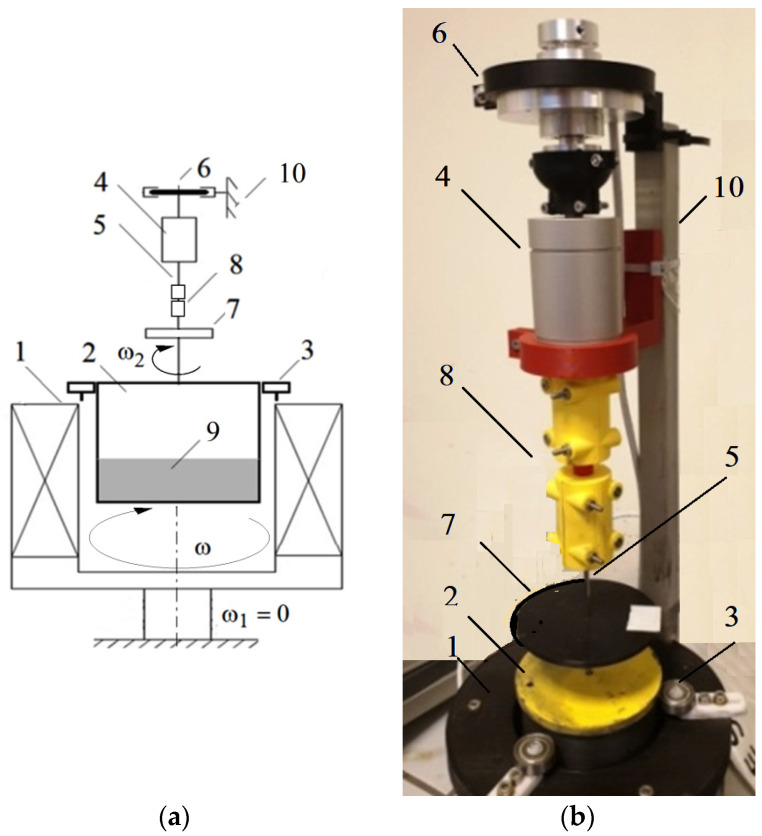
HCMR test stand: (**a**) construction scheme: 1—stator of a three-phase induction motor, 2—beaker, 3—beaker bearing, 4—torque meter, 5—output shaft, 6—brake, 7—rotational speed meter, 8—clutch, 9—MR fluid, 10—frame; (**b**) view.

The scheme of the HCMR test stand is presented in Figure 6. In order to simplify the construction of the test stand, the input shaft was immobilized. A rotating magnetic field with variable rotation speed *ω* was obtained using the winding stator of a three-phase induction motor cooperating with a frequency converter. A rotating vessel with a diameter of 80 mm was mounted in the stator of a three-phase motor using ball bearings positioned around its circumference. This setup allowed observation of the movement of the MR fluid contained in the vessel. A laser angular velocity meter was used to measure the angular velocity. Changes in the torque *M*_2_ on the HCMR output shaft were induced using an internally designed brake and measured using a torque meter. The angular velocity *ω*_2_ and torque *M*_2_ were registered at regular intervals with the use of a PC with specialized software.

Data regarding the components of the test stand are presented in Table 4.

### 2.5. Phenomena Occurring during Operation of the Hydraulic Clutch with MR Fluid

**Figure 7:**
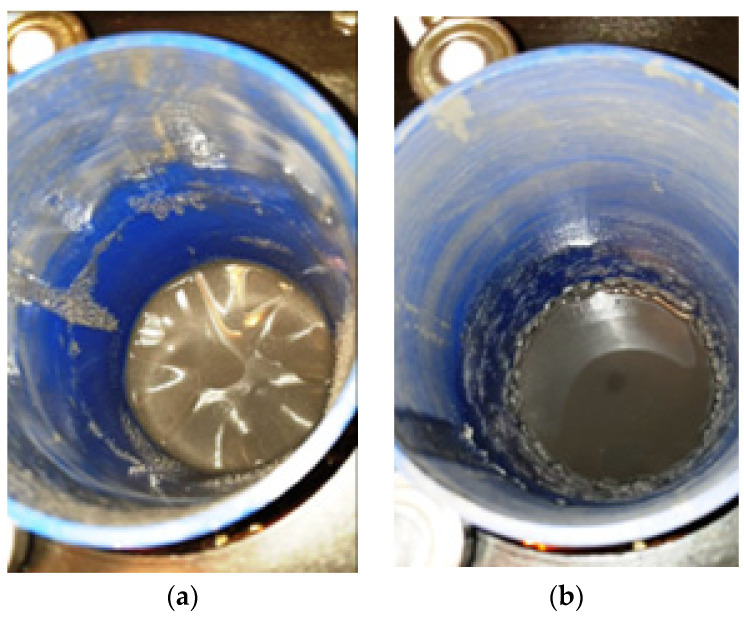
View of the B3 fluid surface at HCMR start-up for *ω* = 240 rad/s: (**a**) wrinkles, (**b**) ring of swirling fluid.

**Figure 8:**
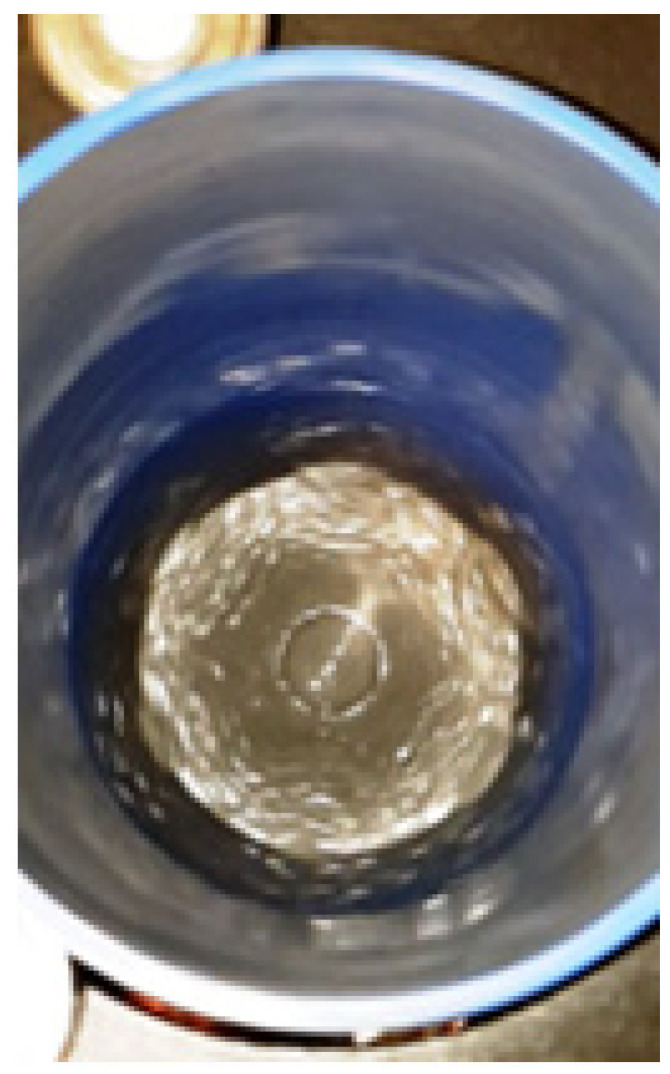
View of a hexagon formed by the rotation of the MR fluid for B3 fluid and *ω* = 240 rad/s.

During the tests of the operation of the clutch with a rotating magnetic field with fluids A and B, it was noticeable that after generating a rotating magnetic field with a constant angular velocity *ω*, the beaker was initially stationary. In the first seconds after turning on the rotating magnetic field, wrinkles appeared on the surface of the fluid (Figure 7a). If the liquid was not well mixed, a rotating ring of solid particles sometimes appeared on the walls of the beaker (Figure 7b). The emergence of wrinkles on the surface of the MR fluid, depicted in Figure 7, suggests the influence of surface tension forces at the interface between two mediums: the MR fluid with magnetic properties and the non-magnetic air [33]. After a while, the fluid at the bottom of the beaker started to rotate with the beaker, assuming a hexagonal shape (Figure 8). Polygon-shaped deformations can also be observed in single-phase fluids [41]. Similar regular deformations during rotation also occur for gases. Such a phenomenon was noticed at Saturn’s north pole on space probe missions such as Voyager 1, Voyager 2 and Cassini [42]. The occurrence of deformations is linked to various factors, including the emergence of secondary flows propelled by centrifugal force, meridional circulation within the fluid, and the development of Görtler vortices as the fluid flows along the walls [43,44].

**Figure 9:**
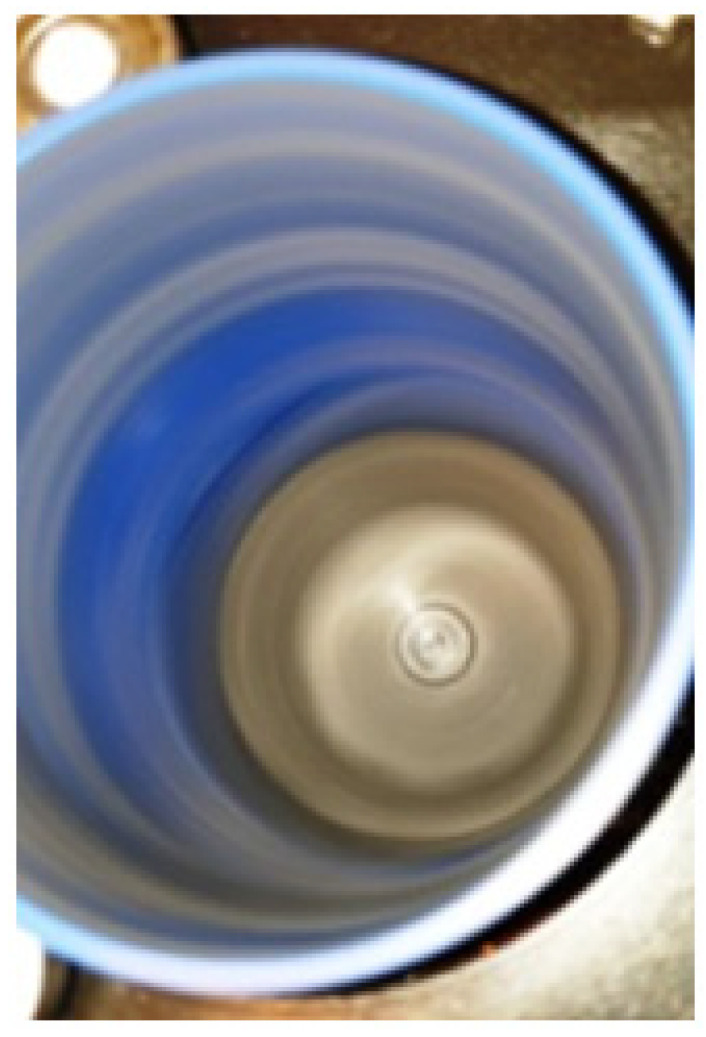
View of the potential vortex in fluid B2.

**Figure 10:**
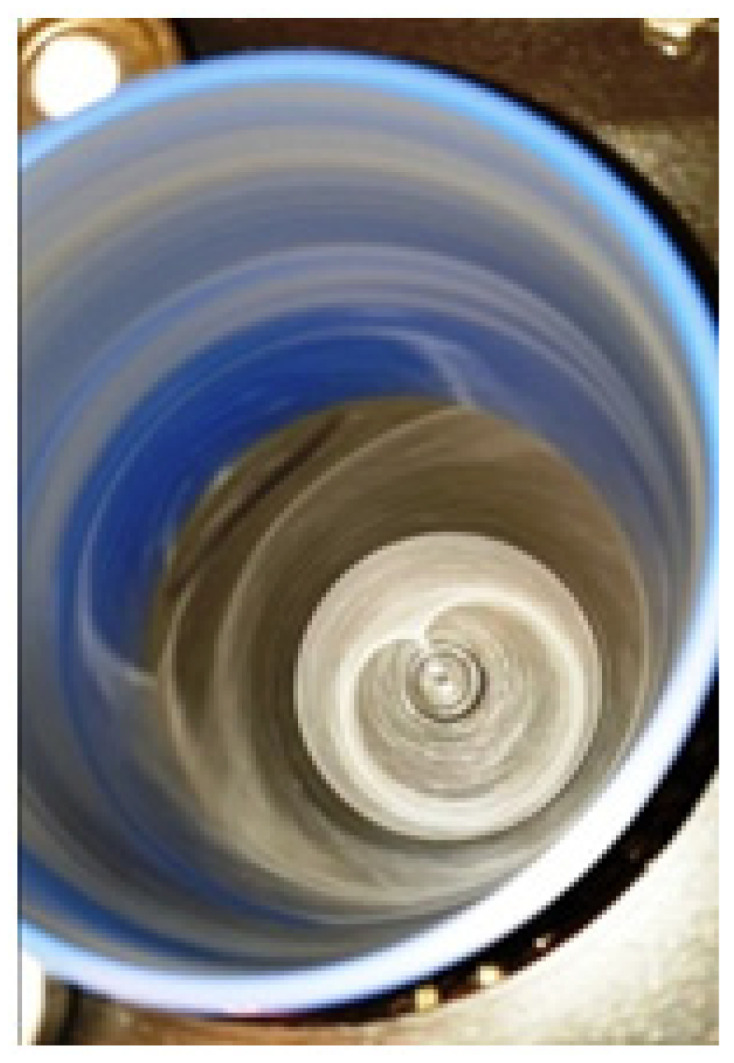
View of the B2 fluid spread evenly on the beaker walls.

After the beaker’s rotation speed *ω*_2_ increased to the maximum value *ω*_2*max*_, the free surface of the fluid in the beaker was arranged as for a potential vortex, assuming a parabolic shape (Figure 9). The maximum rotation speed of the beaker *ω*_2*max*_ is always lower than the angular rotation speed of the magnetic field *ω*. Decelerating the beaker in the presence of a rotating magnetic field caused a decrease in the rotational speed *ω*_2_ of the beaker and an increase in the value of the braking torque *M*. When the beaker reached the maximum centrifugation speed and the magnetic field was turned off, the fluid rapidly spread onto the walls of the beaker (Figure 10).

### 2.6. Test Results of the Characteristics of the Hydraulic Clutch with MR Fluid

**Figure 11:**
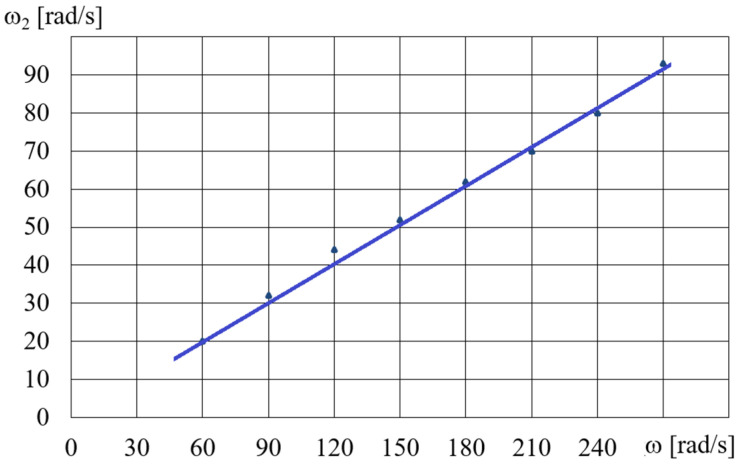
Dependences of angular velocity *ω*_2_ on angular velocity *ω* for the fluid B5 and fluid volume *V* = 75 cm^3^.

**Figure 12:**
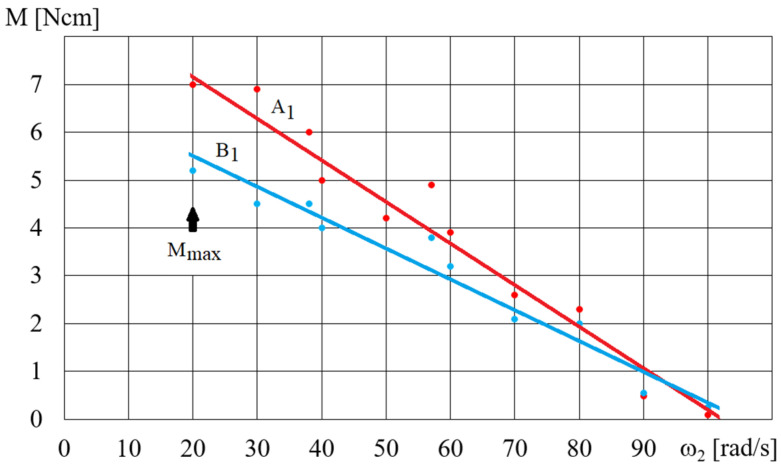
Dependence of the torque *M* on the angular velocity *ω*_2_ for fluids A1 and B1, *ω* = 300 rad/s, *V* = 75 cm^3^.

**Figure 13:**
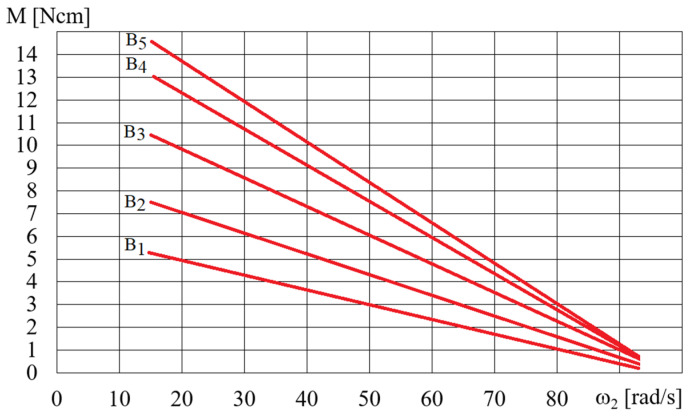
Dependence of the torque *M* on the angular velocity *ω*_2_ for fluids B1–B5 for *ω* = 300 rad/s, *V* = 75 cm^3^.

The HCMR characteristics were tested for a predetermined volume, *V*, of the MR fluid in the beaker. Figure 11 shows a diagram of the dependence of the angular velocity *ω*_2_ of the HCMR output shaft on the angular velocity of the magnetic field rotation *ω*, when the beaker contained the B5 fluid and was not decelerated. It can be observed from Figure 11 that in the absence of braking torque there was a linear relationship between the angular velocity *ω*_2_ of the HCMR output shaft and the rotational speed of the magnetic field *ω*. The slip *s* = (*ω − ω*_2_)/*ω* was almost constant and its average value was 0.66. For instance, in Figure 12 and Figure 13, the relationship between torque *M* and angular velocity of the output shaft *ω*_2_ is illustrated for fluids A and B. The vessel was gradually decelerated using a friction brake at *ω* = 300 rad/s. Figure 12 shows the measurement points and approximation lines. Figure 12 shows that as the torque *M* decreases, the angular velocity *ω*_2_ increases linearly. Higher torque values occur for fluid A1 than for fluid B1, which appears to be associated with the fact that the smaller particle diameter resulted in a higher concentration of Fe in fluid A1 than in fluid B1, and the viscoplastic properties of fluid A1 contributed to increased adhesion to the vessel walls compared to fluid B1. Figure 13 shows the dependences of *M* on *ω*_2_. In order to increase the readability of Figure 13, especially for higher values of angular velocity *ω*_2_, the measurement points are omitted and only the approximating lines are shown. The influence of the mass of iron contained in the MR fluid on the dependence of the torque *M* on the angular velocity *ω*_2_ is also clearly visible in Figure 13. The greater the mass fraction *φ*, and therefore the mass of iron in the fluid B in the beaker, the greater the torque *M*.

**Figure 14:**
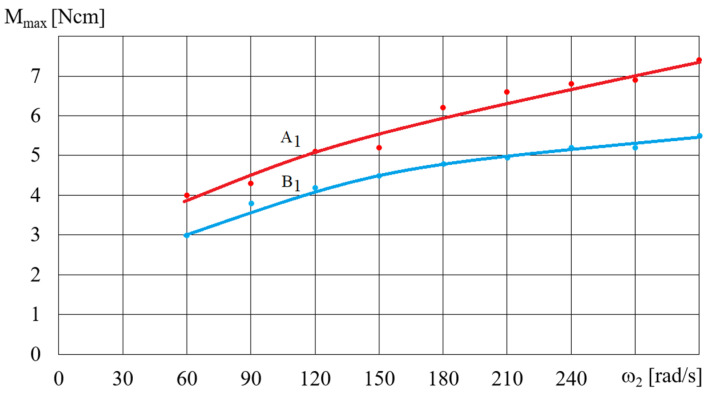
Dependence of the torque *M* on the angular velocity *ω*_2_ for fluids A1 and B1 for *ω* = 300 rad/s, *V* = 75 cm^3^.

**Figure 15:**
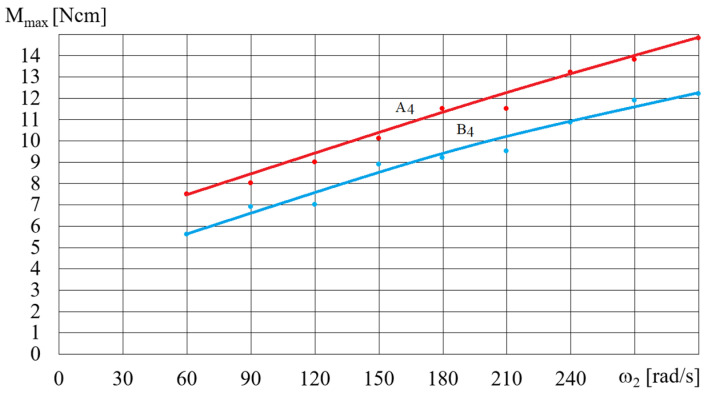
Dependence of the torque *M_max_* on angular velocity *ω* for fluids A4 and B4, *V* = 75 cm^3^.

**Figure 16:**
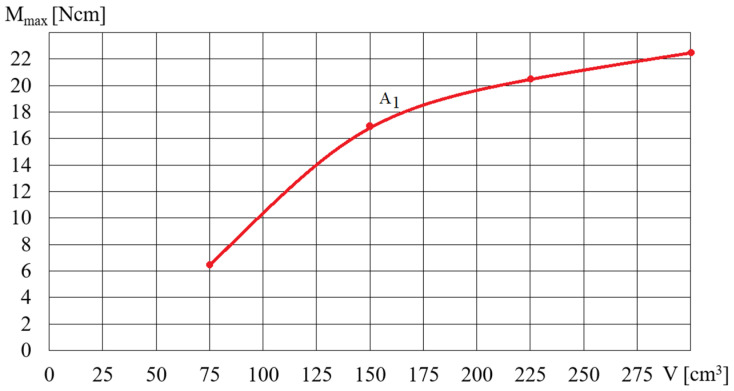
Dependence of the torque *M_max_* on the volume *V* of the fluid A1 for *ω* = 200 rad/s.

Figure 14 and Figure 15 show the dependence of the torque *M_max_* (shown in Figure 12) on the angular velocity for selected fluids A and B, depending on the rotational speed of the magnetic field *ω*. The dependence of the torque *M_max_* on the angular velocity *ω* (presented in Figure 14 and Figure 15) for selected A and B fluids increased: the higher the angular velocity *ω*, the smaller the increase in *M_max_*. Figure 16 shows the dependence of *M_max_* on the volume *V* of the A1 fluid placed in the beaker, for a magnetic field rotational speed of *ω* = 200 rad/s. Figure 16 shows the significant influence of the mass of iron contained in the MR fluid on the dependence of the torque *M_max_* on the volume *V* of the MR fluid. It should be noted that the dependence of the torque *M_max_* on the volume *V* of the MR fluid is not linear but regressive.

## 3. Characteristics of the Hydraulic Clutch with MR Fluid

### 3.1. Operating Method of the Hydraulic Clutch with MR Fluid

**Figure 17:**
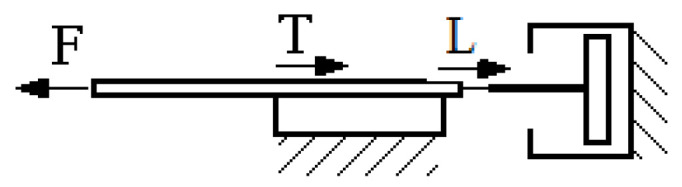
Viscous-plastic model of Bingham’s fluid: *F*—force acting on particles, *T*—friction force, *L*—viscous force.

Conducting a theoretical analysis of HCMR operation poses challenges due to the presence of magnetic forces alongside the forces typically encountered in hydraulic clutches during their operation. Furthermore, the interplay of these forces induces localized changes in density through the phase separation of the MR fluid. The reduction in the angular velocity *ω*_2_ of the HCMR output shaft as the torque value increases takes place within the rotating magnetic field. This decrease primarily stems from the combined effects of centrifugal, gravitational, and magnetic forces. The decrease is also slightly dependent on other forces, such as intermolecular forces, buoyancy, and surface tension. The observation that the MR fluid in a rotating beaker, when subjected to a magnetic field, takes on a free surface resembling a paraboloid (Figure 9) and subsequently spreads onto the beaker walls after the magnetic field is deactivated (Figure 10), provides evidence that the radial magnetic forces acting on solid particles of the MR fluid are smaller but comparable in magnitude to centrifugal forces. The influence of gravity is not significant due to the small amount of fluid in the vessel and therefore its low level. During the operation of the HCMR, the magnetic field, the MR fluid, and the beaker move in rotation. The rotating magnetic field attracts solid particles, which in turn transfer the rotational motion to the beaker walls using friction forces due to the forces pressing the liquid against the beaker walls. The attracted particles move in relation to each other, and their movement is determined by Bingham’s viscous-plastic fluid model, as shown in Figure 17 [45].

**Figure 18:**
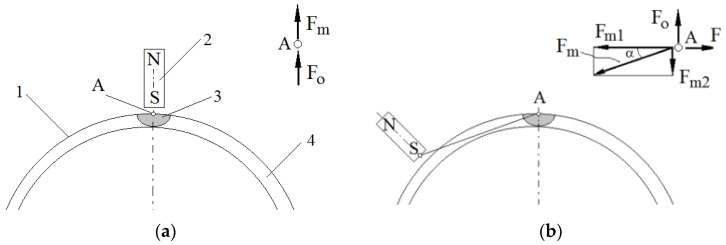
Forces acting on the MR fluid and the beaker during HCMR operation: (**a**) *ω*_2_ = *ω*; 1—beaker wall, 2—magnet, 3—MR fluid pulled by the magnet, 4—a ring of MR fluid; (**b**) *ω*_2_ < *ω*.

In Figure 18 the rotating magnetic field is shown as the pole of a rotating magnet. When the angular velocities of the beaker *ω*_2_ and the field rotation *ω* are equal, the magnetic force acts along the radius of the beaker, because the beaker’s movement is not relative to the pole (Figure 18a). Centrifugal force *F_o_* also acts along the radius. The lack of circumferential force means that the friction force between the MR fluid and the beaker wall does not occur, and there is no torque *M* acting on the fluid and the beaker. When the angular velocity *ω*_2_ of the beaker decreases due to braking, falling below the rotational speed *ω* of the magnetic field, the magnetic pole surpasses the cluster of solid particles. The movement of these particles toward the pole is impeded by viscous forces resulting from tangential stresses in the MR fluid. Then, as presented in Figure 18b, the magnetic force *F_m_* deflects from the radius and can be divided into the circumferential component of the magnetic force *F_m_*_1_ and the radial component of the magnetic force *F_m_*_2_. Such deflection of the magnetic force from the radius is also found during the operation of a powder clutch, on the basis of the observation of the arrangement of solid particle chains in the working gap [8]. The circumferential force *F_m_*_1_ creates a torque acting on the MR fluid ring, while the axial force *F_m_*_2_ reduces the centrifugal force *F_o_*. The reduction of the centrifugal force *F_o_* by the force *F_m_*_2_ is evident from the observation that, following the disappearance of the magnetic field during the beaker’s rotation, the MR fluid influences the walls of the beaker (Figure 10). Simultaneously, the MR fluid moves toward the pole of the magnetic field and is pressed against the beaker wall as a result of the resultant forces *F_o_* − *F_m_*_2_. Simultaneously, the MR fluid, moving towards the magnetic field’s pole, is pressed against the beaker wall due to the resultant force *F_o_* − *F_m_*_2_. This results in a friction force *F* on the beaker walls, generating a torque *M* on the beaker axis. This torque, equal in value but opposite in direction, acts as a braking force on the beaker. Further reduction of the speed of the beaker *ω*_2_ causes an increase in force *F*, whose value is proportional to the difference in rotational speeds (*ω* − *ω*_2_) due to the occurrence of viscous forces. At the same time, it causes a decrease in the centrifugal force *F_o_* pressing the MR fluid ring against the beaker. In the case of lower beaker velocities *ω*_2_, the force (*F_o_* − *F_m_*_2_) is too small to transfer the rotational motion of the MR fluid ring to the beaker movement, as a result of which the HCMR stops operating properly.

### 3.2. Comparison of the Hydraulic Clutch with MR Fluid to Other Clutches

In order to classify the HCMR, it has been compared to other clutches which utilize a magnetic field, such as induction clutches, eddy current clutches, magnetic particle clutches, viscous clutches with MR fluid, electromagnetic friction-type clutches, and magnetic clutches. The structure of HCMR is similar to induction clutches, while its operating principle is similar to magnetic clutches with electromagnets. It can also be regarded as a combination of a viscous clutch, where shear stresses in the MR fluid are altered using a magnetic field, and a hydrodynamic clutch in which a magnetic field acts on the fluid instead of blades. It should be noted that if the MR fluid does not completely fill the beaker in which it is located, its movement along the walls of the beaker under the influence of centrifugal force and the magnetic field will affect the performance of the clutch due to the changing friction surface. This feature distinguishes the HCMR from other magnetic clutches.

**Figure 19:**
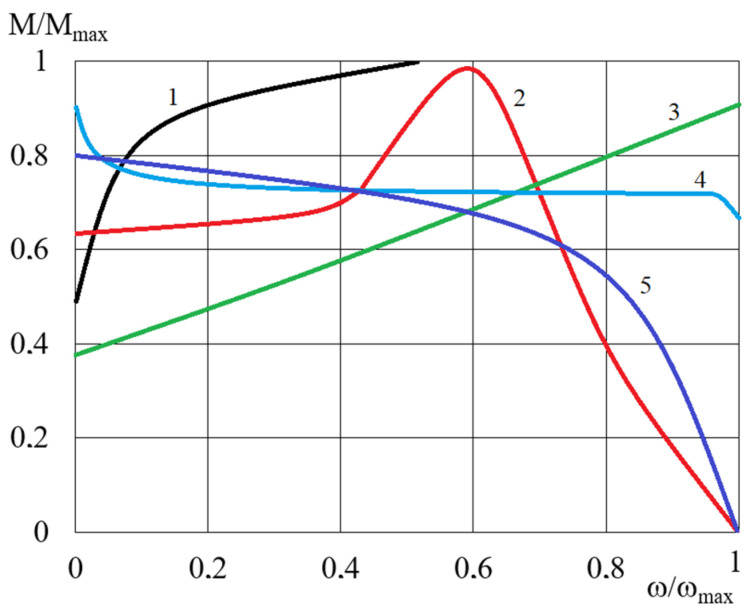
Characteristics of clutches using the influence of a magnetic field: 1—electromagnetic friction-type clutches [8], 2—induction clutches [45], 3—viscous clutches with MR fluid [47], 4—magnetic particle clutches [8], 5—eddy current clutches [8].

Based on the data from publications [8,46,47], Figure 19 shows characteristics of clutches which use the influence of a magnetic field. The comparison of Figure 19 and Figure 12 shows that the HCMR’s characteristics are similar to the characteristics of eddy current clutches. Therefore, the proposed new clutch which uses MR fluid and a rotating magnetic field can be applied to stationary power transmission systems in a similar manner to eddy current clutches. The advantages of clutches with a rotating magnetic field, along with other clutches using magnetic fields operating in drive systems, include controllability, high durability, and the absence of a rigid connection between the driving and driven parts. This last characteristic makes the drive system shafts insensitive to misalignment.

## 4. Conclusions

On the basis of the considerations regarding HCMR, the following general conclusions have been drawn:

(1) The literature review shows that a rotating magnetic field can cause macroscopic rotation of the MR fluid with a rotational speed lower than the rotational speed of the magnetic field. Whether such a movement will occur and what its direction will be depends on a number of factors related to the properties of the MR fluid and the operating conditions. Due to the complexity of the phenomena occurring, no single model has been developed so far to explain the causes of rotation of the MR fluid in a rotating magnetic field. Most authors believe that the shape of the model is significantly impacted by the inhomogeneities in the MR fluid affected by the rotating magnetic field.
(2) The impact of the magnetic field on the rheological properties of MR fluids with varying sizes of iron particles, assessed using a customized rheometer with a magnetic device, can be accurately determined for MR fluids with iron particles having diameters not exceeding 6.5 μm. Iron particles with a larger diameter exhibit a stronger attraction towards the poles of the electromagnet situated outside the measuring gap. This results in a reduction of the iron content in the vicinity of the rheometer spindle, thereby limiting the influence of the magnetic field on the measured torque. Therefore, other measurement methods should be sought for MR fluids with larger iron particles.
(3) In the absence of a load on the HCMR output shaft, the angular velocity of the beaker containing the MR fluid exhibits a linear dependence on the angular velocity of the magnetic field. However, during shaft braking, the torque transmitted by the clutch increases with higher angular velocity of the magnetic field and lower angular velocity of the beaker. The highest torque occurs for MR fluids with the highest iron content and solid particles with dimensions ranging from 3.5 to 6.5 μm.
(4) The characteristics of HCMRs are similar to the characteristics of eddy current clutches and result mainly from the combined action of magnetic and centrifugal forces on the MR fluid. The solid particles of the MR fluid are attracted by the rotating magnetic field and simultaneously pressed against the walls of the beaker, mainly due to the influence of centrifugal forces. The rotational motion of the beaker is induced by the friction force generated due to the applied pressure. The magnitude of the friction force, which propels the rotation of the beaker, increases with the greater difference between the rotational speed of the magnetic field and that of the beaker. The viscous-plastic properties of the MR fluid cause the solid particles of the MR fluid to be slower than the rotation of the magnetic field.
(5) It is finally remarked here that further research regarding HCMRs should aim to design, construct and test a clutch prototype and to select MR fluids with the optimal size and shape of solid particles so that the proposed system can be applicable to several types of the hydraulic control systems in real environments subject to various uncertainties such as time-varying temperature.

## Figures and Tables

**Table 1:** Names and composition of the in-house manufactured MR fluids.

Name	Fe Particle Size	Base Fluid
A	From 3.5 μm to 6.5 μm	Silicon oil OL.111
B	From 100 μm to 150 μm	Silicon oil OL.111

**Table 2:** The weight concentration ratio in the tested MR fluids.

Symbol	Density [g/cm^3^]	Weight Concentration Ratio of Solid Particles *φ* [%]
1	1.69	0.50
2	2.01	0.60
3	2.30	0.67
4	2.56	0.72
5	2.79	0.75

**Table 3:** Dimensions of the magnetic device for the rheometer Brookfield DV2T.

Parameter	Value
Spindle radius RV-07 (7)	2.1 mm
Internal radius of the tank opening	3.25 mm
Maximum spindle immersion depth	35 mm
Length of the electromagnet core	276 mm
Cross-section of the electromagnet core	24 mm × 12 mm
Wire diameter and number of turns of the electromagnet coil	0.25 mm × 850
Spindle angular velocity range	2 rad/s–10 rad/s
MR fluid temperature during testing	25 °C

**Table 4:** Components of the test stand.

Component	Designation	
Frequency converter	LG SV008iC5-1F (LS ELECTRIC Co., Ltd., Anyang-si, Republic of Korea)	
Engine stator	230 V	0.75 kW
Brake	MH1	
Temperature sensor	NTC 215	
Angular velocity meter	MeasureMe MT522	
Torque gauge	MT1	

## Data Availability

The original contributions presented in the study are included in the article, further inquiries can be directed to the corresponding author.
